# Bacteria in Dairy Products in Baggage of Incoming Travelers, Brazil

**DOI:** 10.3201/eid2011.131422

**Published:** 2014-11

**Authors:** Cristiano Barros de Melo, Marcos Eielson Pinheiro de Sá, Antonizete dos Reis Souza, Anapolino Macedo de Oliveira, Pedro Moacyr Pinto Coelho Mota, Paulo Ricardo Campani, Janaína Oliveira Luna, Sérgio Cabral Pinto, Fábio Fraga Schwingel, Concepta McManus, Luiza Seixas

**Affiliations:** University of Brasília, Brasília, Brazil (C.B. de Melo, A.R. Souza, C. McManus, L. Seixas Author affliliations:);; Ministry of Agriculture, Livestock and Food Supply (MAPA), Brasília, Brazil (M.E.P. de Sa);; MAPA, Galeão Airport, Rio de Janeiro, Brazil (P.R Campani);; MAPA, Guarulhos Airport, São Paulo, Brazil (J.O. Luna);; MAPA, Confins International Airport, Belo Horizonte/Confins, Brazil (S. Cabral Pinto);; MAPA, Brasilia International Airport (BSB), Brasília, Brazil (F.F. Schwingel);; MAPA, Pedro Leopoldo, Brazil (A.M. de Oliveira, P.M.P.C. Mota)

**Keywords:** Zoonoses, tuberculosis, bovine, brucellosis, paratuberculosis, foodborne, *Brucella*, *Mycobacterium bovis*, and *Mycobacterium avium* subsp. *paratuberculosis*, MAP, Brazil

**To the Editor:** International air travel can lead to the rapid global dissemination of infectious agents. Unlike products and byproducts of animal origin imported between countries under agreements that legally establish sanitary standards, products introduced into a country illegally or irregularly do not follow specific standards and can come from any source, thereby posing a risk to the health status of a country. Animal products transported clandestinely in baggage can contain infectious agents harmful to animal and human health (*1*–*4*). We investigated *Brucella* spp., *Mycobacterium bovis*, and *Mycobacterium avium* subsp. *paratuberculosis* (MAP) in dairy products seized from baggage of passengers on flights at the 2 main international airports (Guarulhos Airport, São Paulo, and Galeão Airport, Rio de Janeiro), in Brazil.

During 2010–2011, 12 missions were instigated by the International Agriculture Surveillance (VIGIAGRO/MAPA) in airports to detect and seize unauthorized dairy products carried by passengers; 195 products were collected from multiple flights from different destinations. Baggage was scanned by using an x-ray machine and, on detection of a product, was opened by the owner in the presence of a federal agriculture inspector. To avoid contamination, the products were not opened and were sent to the designated Ministry of Agriculture, Livestock and Food Supply Laboratory in their original packaging. All seized products were packed according to the International Air Transport Association standards (*5*) and transported by commercial aviation with official monitoring to the laboratory.

After completing real-time quantitative PCR (Promega, Madison, WI, USA) using TaqMan technology (Life Technologies, Darmstadt, Germany), we extracted DNA directly from the sample (*6*,*7*). The technique for the detection of MAP and eryD *Brucella* (except strain 19 *Brucella abortus*) and also using the region RD4 to detect *M. bovis* were proposed by Irange et al. (*8*). To detect *M. bovis*, we used the primers *M. bovis*-88-F (5′-CGC.CTT.CCT.AAC.CAG.AAT.TG-3′), *M. bovis*-88-R (5′-GGA.GAG.CGC.CGT.TGT.AGG-3′) and to detect *Brucella,* we used Bru-Eri-Taq-92-F (5′-GCC.ACA.CTT.TCT.GCA.ATC.TG-3′) and Bru-Eri-Taq-92-F (5′-GCG.GTG.GAT.AAT.GAA.ATC.TGC-3′).

We analyzed 35 containers of dulce de leche, a caramelized milk paste confection, from Argentina (n = 30), Angola (n = 1), and Uruguay (n = 4). We tested all specimens for *Brucella* spp. and MAP, and 32 for *M. bovis*. We detected MAP in 1 specimen from Argentina and 1 from Uruguay, *Brucella* spp. in 3 specimens from Argentina and 1 from Uruguay, and *M. bovis* in 1 specimen from Argentina.

Three containers of liquid milk from the United States were collected and analyzed for the presence of MAP; 2 were analyzed for *M. bovis* and *Brucella.* *Brucella* was detected in 1 specimen. Five containers of powdered milk were seized: 2 from Chile, 2 from Angola, and 1 from Portugal. *Brucella* was detected in 1 container from Chile; *Brucella* and *M. bovis* were found in 1 container from Angola. Four containers of yogurt were seized, 1 each from the United States, China, Angola, and South Africa. MAP was detected in those from Angola and South Africa, and the yogurt from South Africa also showed *Brucella*.

We analyzed samples from 147 cheeses that were confiscated from baggage owned by travelers from 21 countries, mainly from Italy (24.5%), Portugal (22.4%), and France (14.3%). *M. bovis* was identified in 18 (17.5%) cheeses collected from Italy, Portugal, Spain, the United States, the Netherlands, Lebanon, Morocco, and Norway. MAP was amplified in specimens from 13 cheeses from Spain, United States, Iraq, Israel, Norway, Peru, France, and Portugal, the last 2 countries showed the highest occurrence. *Brucella* was detected in 62 of the cheeses analyzed, which originated in Bolivia, Chile, Iraq, Lebanon, and Morocco (n = 1 in each country), Netherlands, Israel, and Norway (n = 2 in each country), Turkey and Spain (n = 3 in each country), United States, France and England (n = 4 in each country), Portugal (n=10), and Italy (n = 23).

Both *M. bovis* and *Brucella* were detected in 13 (8.8%) cheeses (1 each from Spain, Netherlands, Morocco, and Norway; 4 from Portugal, and 5 from Italy); *Brucella* and MAP were detected in 4 (2.7%) cheeses (Spain, France, Portugal, and Iraq). Co-amplification of the 3 genes (*Brucella* + MAP + *M. bovis*) occurred in 3 (2%) cheeses (United States, Norway, and Portugal). Among the cheeses analyzed, 84 (57.1%) contained isolates that amplified >1 of the genes for the 3 bacteria examined.

Of the 166 dairy products analyzed, *Brucella* was detected in 70 (42.1%). Cheeses were the most seized products (n = 121) and had the highest number of *Brucella*-positive results (62/121[51.2%]). *Brucella* was detected in dairy products that originated in Argentina, Spain, France, Iraq, Israel, Italy, Lebanon, Portugal, and Turkey; it was detected in 4 (21%) of the 19 cheeses from France and in 3 of the 4 (75%) cheeses that originated in Spain. *M. bovis* was detected in dulce de leche from Argentina, powdered milk from Chile, and in cheeses from Spain, Netherlands, Italy, Lebanon, Morocco, Norway, and Portugal.

Bacteria can be introduced into a country through contaminated animal products that are brought across borders illegally. The risk may be even greater when these products are carried in passengers’ baggage on international flights because of the growing number of international travelers and the wide range of origins of these passengers. Greater attention should be given to agricultural surveillance at airports to mitigate the risk for introduction of these products.
